# Centrosome Remodelling in Evolution

**DOI:** 10.3390/cells7070071

**Published:** 2018-07-06

**Authors:** Daisuke Ito, Mónica Bettencourt-Dias

**Affiliations:** Instituto Gulbenkian de Ciência, Rua da Quinta Grande 6, 2780-156 Oeiras, Portugal

**Keywords:** centrosome, centriole, spindle pole body, SPB, PCM, evolution

## Abstract

The centrosome is the major microtubule organizing centre (MTOC) in animal cells. The canonical centrosome is composed of two centrioles surrounded by a pericentriolar matrix (PCM). In contrast, yeasts and amoebozoa have lost centrioles and possess acentriolar centrosomes—called the spindle pole body (SPB) and the nucleus-associated body (NAB), respectively. Despite the difference in their structures, centriolar centrosomes and SPBs not only share components but also common biogenesis regulators. In this review, we focus on the SPB and speculate how its structures evolved from the ancestral centrosome. Phylogenetic distribution of molecular components suggests that yeasts gained specific SPB components upon loss of centrioles but maintained PCM components associated with the structure. It is possible that the PCM structure remained even after centrosome remodelling due to its indispensable function to nucleate microtubules. We propose that the yeast SPB has been formed by a step-wise process; (1) an SPB-like precursor structure appeared on the ancestral centriolar centrosome; (2) it interacted with the PCM and the nuclear envelope; and (3) it replaced the roles of centrioles. Acentriolar centrosomes should continue to be a great model to understand how centrosomes evolved and how centrosome biogenesis is regulated.

## 1. Introduction

In 1887, the German biologist Theodor Boveri first described and named the structure at the pole of a mitotic spindle as “centrosome” [1]. The centrosome is the major microtubule organizing centre in eukaryotic cells, playing critical functions for cell division, motility and signalling. In animal cells, the canonical centrosome is composed of centrioles surrounded by the pericentriolar matrix (PCM), an electron-dense proteinaceous matrix that nucleates microtubules [2]. The centriole is a cylinder-like structure made of nine triplet microtubules arranged in nine-fold symmetric configuration. Importantly, centrioles are converted to basal bodies and serve another function to nucleate the formation of cilia and flagella, which are essential for signalling and movement. Centrioles/basal bodies and cilia/flagella are present in all major eukaryotic groups, suggesting that these are ancestral structures, that is, cells of the last common ancestor of eukaryotes already had cilia for motility [3]. It is proposed that centrosomes evolved by internalization of the centriole/basal bodies and coordinating cell division in close association with the nucleus [4,5].

Fungi and amoebozoa show great structural diversity in their centrosomes, suggesting very distinct remodelling during evolution [6,7]. Within these groups, the yeasts and slime moulds have no centriole and possess highly remodelled acentriolar centrosome, the spindle pole body (SPB) and the nucleus-associated body (NAB), respectively [8,9]. Although the structure, function and biogenesis of the divergent centrosomes have been intensively studied so far, it still remains elusive how these structures evolved. In this review, we compare the structures and molecular components in centriolar and acentriolar centrosomes and speculate how these remodelled centrosomes evolved.

## 2. Canonical and Diverged-Centrosomes in Animal, Fungi and Amoebozoa

**Figure 1:**
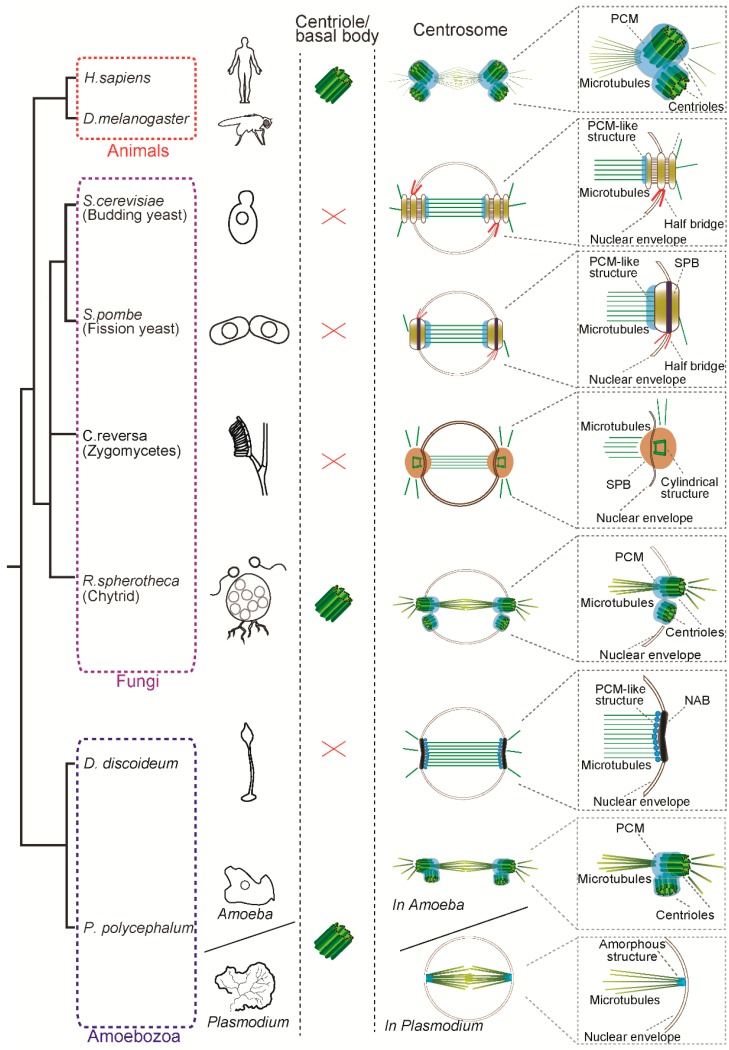
Structure of the centrosome in mitosis in animals, fungi and amoebozoa. Animals and basal fungi (chytrids) have a centriole/basal body and a canonical centrosome composed of a pair of centrioles surrounded by pericentriolar matrix (PCM), which anchors and nucleates microtubules. In contrast, the yeasts such as *S. cerevisiae* and *S. pombe* have a centriole-less centrosome called the spindle pole body (SPB). Another fungus *C. reversa* has a distinct SPB containing a cylindrical structure reminiscent of a centriole. In the Amoebozoa, while *D. discoideum* has a centriole-less nucleus-associated body (NAB), *P. polycephalum* has two modes of centrosomes: animal-like centriolar centrosome in the amoeba phase and an amorphous structure devoid of centriole at the pole in the plasmodium phase. Note that size of the centrosomes and spindle are not depicted to scale in this figure.

We focus on centrosomes in the eukaryotic group Opisthokonta, which includes animal and fungi and the sister group Amoebozoa, forming the supergroup Amorphea [10,11]. This is the group where the cell and molecular biology of centrosomes is better described and their centrosomes show much diversity in structures and composition (Figure 1). The SPB is a multi-layered structure composed of a centriole-less scaffold that recruits γ-tubulin [12,13]. Yeasts undergo closed mitosis, in which the nuclear envelope remains intact during cell division. In the budding yeast *Saccharomyces cerevisiae* and the fission yeast *Schizosaccharomyces pombe*, the SPB acts as an MTOC and is required for cell division. The SPB is embedded in the nuclear envelope and nucleates spindle microtubules inside the nucleus as well as astral microtubules toward the cytoplasmic side (Figure 1). In both species, the structure attached to the nuclear envelope, called the half-bridge, plays an important role in SPB duplication (Figure 1) [8,14]. Similar to the yeasts, the closely-related zygomycete fungal species *Coemansia reversa* lacks flagella and centriole and has a spindle pole body which acts as an MTOC [15]. Interestingly, electron microscopy revealed that its SPB contains a cylindrical ring structure (68–71 nm in height and 80–100 nm in width), which has nine microtubules and appears to be *reminiscent* of a centriole. Although the molecular composition of the structure is not known yet, it might be a “degenerated” centriole associated with the SPB. It is worth noting that not all the fungi have an SPB: the zoosporic, basal fungi such as chytrids (e.g., *Rhizophydium spherotheca*) have both a centriole-containing centrosome and flagella [16]. As shown in Figure 1, this species undergoes closed mitosis similarly to the yeasts and the centrioles reside in the fenestrae in the nuclear envelope (NE) to form mitotic spindles within the nucleus.

In the *Amoebozoa*, the centrosome structures have been well described by electron microscopy in the two important model organisms—the slime moulds *Dictyostelium discoideum* and *Physarum polycephalum.* The centrosome in *D. discoideum* is called the nucleus-associated body (NAB), which is an acentriolar three-layered match-box shaped structure surrounded by a microtubule-nucleating corona [9]. Like the yeast SPB, the NAB is embedded in the NE and nucleates spindle microtubules inside the nucleus during mitosis (Figure 1) [17]. In contrast, *P. polycephalum* and the related species (*P. flavicomum* and *Echinostelium minutum*) show two distinct modes of cell division and two types of centrosomes. During its life cycle, *P. polycephalum* exists as a uninucleate amoeba and as a syncytial plasmodium containing many nuclei [18]. During the uninucleate amoeba phase, mitosis is open and the centrosome contains a pair of centrioles like animals [19,20,21]. During the plasmodial phase, the organism switches to closed mitosis and the intranuclear microtubules are nucleated from the amorphous structure which contains γ-tubulin [22,23,24]. Although it is not known how this change during differentiation is regulated, the transition from centriolar to acentriolar transition is correlated with loss of flagella and it is possible that the acentriolar and closed mitosis might be beneficial during the plasmodial phase.

Parsimoniously, it is likely that the common ancestor of animals, fungi and amoebozoa had centrioles to form cilia and a centriole-containing centrosome with a PCM structure and parallel to the loss of centriole, the remodelled acentriolar centrosomes were acquired in some species such as yeasts and *D. discoideum*. How did these centrosomes evolve? In the further sections, we focus on the comparison between animal centrosomes and yeast SPB, in which the structures, molecular components and regulators are well-characterised to date. Despite the structural differences described above, there is evidence suggesting that the centriolar centrosomes and the SPB are homologous structures, that is, common molecular components and regulators involved in their biogenesis. In the next section, we describe the molecular composition of the centrosome and regulators in animal and yeast centrosomes.

## 3. Common Components in Centrosome

Many of the components of centrosomes have been identified by genetic and biochemical approaches in animal and yeast centrosomes to date (reviewed in [13,25,26]). In this review, we briefly describe the core components and focus to highlight what is common and what is different.

**Figure 2:**
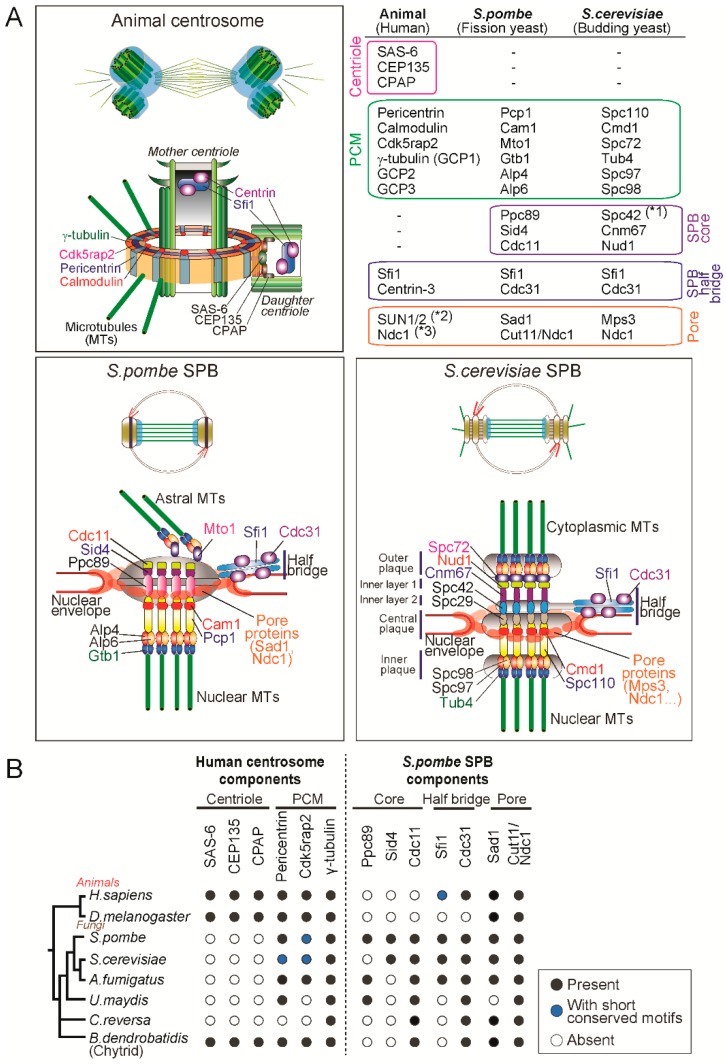
The molecular components of animal centrosomes and yeast SPBs. (**A**) Mapping of molecular components in the animal centrosome and budding and fission yeast SPBs. (*1. Spc42 seems functionally analogous to Ppc89 [61], *2,3. The protein is conserved but not known to be implicated in centrosome functions.) The illustrated SPB structure was adapted and modified from [13]. (**B**) Phylogenetic distribution of centrosome components in animals and fungi. We searched for orthologues of components of the human centrosome localizing to centrioles and PCM and the fission yeast SPB components by using reciprocal pairwise sequence-based (BLASTP and phmmer) and domain-based (hmmsearch) methods [87,88]. Black circles represent the presence of orthologues; blue circles indicate that previous studies showed the presence of a protein with short conserved motifs [47,56,60] although we failed to identify it by the above-mentioned computational methods; white circles indicate no detectable orthologue ([89] and our unpublished results).

In animals, when a daughter centriole (procentriole) starts forming close to the mother centriole, the first assembled is a scaffolding structure called the cartwheel: ninefold symmetric and composed of SAS-6, CEP135, STIL [27,28,29,30,31]. Cartwheel formation is followed by centriole elongation through the deposition of centriolar microtubules which is dependent on components such as CPAP [32,33,34]. The major components of the PCM, CDK5RAP2 and pericentrin, are recruited around the centrioles, which then recruit and activate γ-tubulin ring complexes (γ-TuRC), leading to the formation of a competent matrix for microtubule nucleation and mitotic spindle assembly [35,36,37,38,39,40,41,42]. The γ-TuRC is a lock-washer-shaped ring-like structure composed of the highly conserved γ-tubulin and five γ-tubulin complex proteins (GCP2-6) and acts as a template for microtubule nucleation (reviewed in [43,44,45]). Two molecules of γ-tubulin, GCP2 and GCP3 form a tetrametric γ-tubulin small complex (γ-TuSC) and multiple γ-TuSCs are assembled into an active γ-TuRC promoted by the receptor proteins (such as CDK5RAP2 and pericentrin) and GCP3-6 in a cell cycle-dependent manner [44]. Super resolution microscopy studies revealed that the PCM is not an amorphous material but a layered-structure organized by the proteins with specific configurations [37,38]. In particular, one of the PCM components, pericentrin, is extended radially from the centriole with the C-terminal conserved centrosome-targeting PACT domain [46] located close to the centriole wall and the N-terminus, which interacts with γ-TuRC [47], extending outward as illustrated in the Figure 2A.

The yeast spindle pole body is structurally distinct from animal centrosomes and devoid of centrioles but shares several components. The SPB of *S. cerevisiae* is composed of five layers: the outer, inner and central plaques and the inner layers 1 and 2 (Figure 2). The central plaque is connected to the NE and the outer and inner plaques are the site from which cytoplasmic and nuclear microtubules are nucleated.

The *S. cerevisiae* cells nucleate microtubules using the minimal version of γ-tubulin complex (γ-TuSC) consisting of γ-tubulin/Tub4, GCP2/Spc97 and GCP3/Spc98 [13,44]. Importantly, receptors for the γ-tubulin complex Spc110 and Spc72 sit on the nuclear and cytoplasmic sides of the SPB (Figure 2). Spc110 is a pericentrin orthologue and its N-terminal region interacts with GCP3/Spc98, thereby recruiting γ-TuSC to the nuclear side of SPB [48,49]. At the cytoplasmic side, Spc72, a CDK5RAP2 orthologue, binds to γ-TuSC and nucleate microtubules which are required for proper spindle positioning [50,51]. The central core of the SPB is a hexagonal crystal lattice composed of the scaffold protein Spc42 [52]. It associates with other structural proteins Spc29, Cnm67 and the γ-TuSC receptor Spc110 [53,54,55] (Figure 2).

The main structural component of the half bridge is Sfi1, a large protein containing multiple repeats that binds to the small calcium binding protein Cdc31/centrin3, which has an essential function in SPB duplication [56,57]. The N-terminus of Sfi1is associated with the SPB core and the C-terminus is located at the distal end of the half-bridge [56,58]. This finding led to a structural model for SPB duplication which proposes that the half bridge is elongated by dimerization of Sfi1 at the C-terminus and the new SPB is assembled at the newly formed N-terminus of Sfi1 [8,56]. 

Compared to the *S. cerevisiae* SPB, the *S. pombe* SPB shows a less distinct layered structure but has similar configurations: (1) the SPB is embedded in the NE; (2) nucleates nuclear and cytoplasmic microtubules; and (3) has the half bridge structure (Figure 2A). First of all, unlike *S. cerevisiae* but similarly to animals, *S. pombe* has γ-TuRC components including γ-tubulin/Gtb1 and five GCP proteins (GCP2/Alp4, GCP3/Alp6, GCP4/Gfh1, GCP5/Mod21 and GCP6/Alp16) and nucleates microtubules by forming a ring-like structure (γ-TuRC) (reviewed in [13,43,44]). Importantly, the γ-TuRC receptors Pcp1/pericentrin [59] and Mto1/CDK5RAP2 [60] reside on nuclear and cytoplasmic sides, respectively and promote microtubule nucleation [13,61]. Pcp1 targets not only the γ-TuRC but also Polo-like kinase to the nuclear side of the SPB and is essential for mitotic entry and spindle formation [62]. 

In *S. pombe*, the scaffold proteins Spc42 and Spc29 are not conserved but another protein Ppc89 plays an analogous role at the core of the SPB [61]. Ppc89 is required for localization to the SPB of many of the other components and its overexpression leads to the formation of an enlarged extension of the SPB, suggesting a role as a platform for SPB assembly [63]. It is proposed that Ppc89 connects Pcp1 at the N-terminus and the two structural proteins Sid4 and Cdc11 required for septation initiation network [64,65], at the C-terminus [61] (Figure 2A).

The *S. pombe* half-bridge is also composed of Sfi1-Cdc31 complexes and it is thought that SPB duplication is similarly conserved to *S. cerevisae* (described above), even though the N- and C-termini of the Sfi1 molecule have diverged [13,66,67]. Intriguingly, Sfi1 and its binding partner—centrin-family proteins—are conserved in humans and are shown to localize to the lumen of the centriole [56,68,69]. Despite the conservation, it does not seem that vertebrate Sfi1-centrin is involved in centriole duplication since deletion of centrin genes in chicken DT40 cells [70] or depletion of centrin2 in human cells [71] does not affect duplication, suggesting different roles in animal and yeasts.

Notably, the unique property of the SPB is that it is inserted and anchored in the NE. While *S. cerevisiae* SPB is embedded in the NE throughout the cell cycle [72], *S. pombe* SPB resides in the cytoplasmic side of the nucleus and it is only inserted into the NE during mitosis [73,74]. Although the mode of insertion into the NE is different in the two yeasts, they share molecules involved in the insertion and anchoring. Importantly, the SPB and the NPC (nuclear pore complex) is inserted in the regions of the NE where the INM (inner nuclear membrane) and ONM (outer nuclear membrane) are contiguous (known as the pore membrane) [75]. Moreover, the key player in the insertion of both structures is the conserved transmembrane protein Ndc1 (also known as Cut11 in *S. pombe*) [76,77]. In *S. cerevisiae*, the network of the several SPB proteins including Ndc1, Nbp1, Bbp1 and Mps2 is important for SPB insertion into the NE [78,79]. A recent super resolution microscopy study revealed that Ndc1 and Mps2 form ring-like structures encircling the SPB, indicating these proteins make pore-like structures in the NE (Figure 2) [80]. In *S. pombe*, although it is not known if Ndc1/Cut11 forms a ring around the SPB, another SPB component Sad1 does so (Figure 2) [61]. Sad1 is the conserved SUN-domain protein that is part of the LINC complex connecting cytoskeleton and nucleoskeleton and is required for SPB insertion into the NE [81,82]. Consistently, Mps3, the *S. cerevisiae* orthologue of Sad1, also localises to the periphery of the SPB and interacts with other pore proteins such as Ndc1 [83,84]. This suggests that the two yeasts use analogous machinery to insert the SPB and anchor it at the NE.

To better understand centrosome evolution in animal and fungi, we analysed the conservation of the molecular components of the centrosome, searching for orthologues of the known human centrosome proteins required for centriole assembly (SAS-6, CPAP, CEP135) and the PCM (pericentrin, CDK5RAP2 and γ-tubulin). ln addition, to understand how the SPB originated, we also searched for orthologues of the fission yeast SPB components: the core scaffold proteins (Ppc89, Sid4 and Cdc11) [61,63,64,65], the half-bridge proteins (Sfi1 and Cdc31/centrin3) [56,85] and the pore proteins (Sad1 and Cut11/Ndc1) [61,77,81].

Consistent with previous studies [7,86], the proteins required for centriole biogenesis in animals were not identified in the fungal genomes, with exception of chytrids, which have centrioles (Figure 2B). Although the centriole-like cylinder is present in the *C. reversa* SPB, we did not detect orthologues of the centriole components in its genome, suggesting that the intact building blocks of the centrioles are lost, or the sequences might have evolved at a higher rate. In contrast, the PCM components, pericentrin and CDK5RAP2, together with γ-tubulin, were found in animal and most fungal species analysed (Figure 2B).

When it comes to SPB components, we found that the core proteins such as Ppc89 and Sid4 are conserved only in yeasts but not in chytrids, suggesting that these SPB proteins only appeared after branching into yeasts (Figure 2B). Interestingly, Cdc11 is present in all fungal genomes tested, implying that it was present at the centrosome of the fungal ancestors. The half-bridge localizing Cdc31/centrin3 is highly conserved in most species (except *Drosophila* which lacks centrin3 [5]) but its scaffold Sfi1 was not identified many species in our analysis perhaps as it is very divergent [56]. It is still not clear when the half-bridge and its constituent Sfi1-centrin3 complex emerged. In contrast, the pore proteins were identified in the all the fungal species as well as in animals, suggesting that these played important roles of connecting NE and centrosomes in the animal-fungal common ancestor (to be discussed below). 

Altogether, the phylogenetic distribution of the centrosome components indicates that while centriole components were lost and SPB components were acquired in yeasts, the PCM module including pericentrin and CDK5RAP2 and the connection with NE remained in terms of composition and function.

## 4. Common Regulations in Centrosome Biogenesis

**Figure 3:**
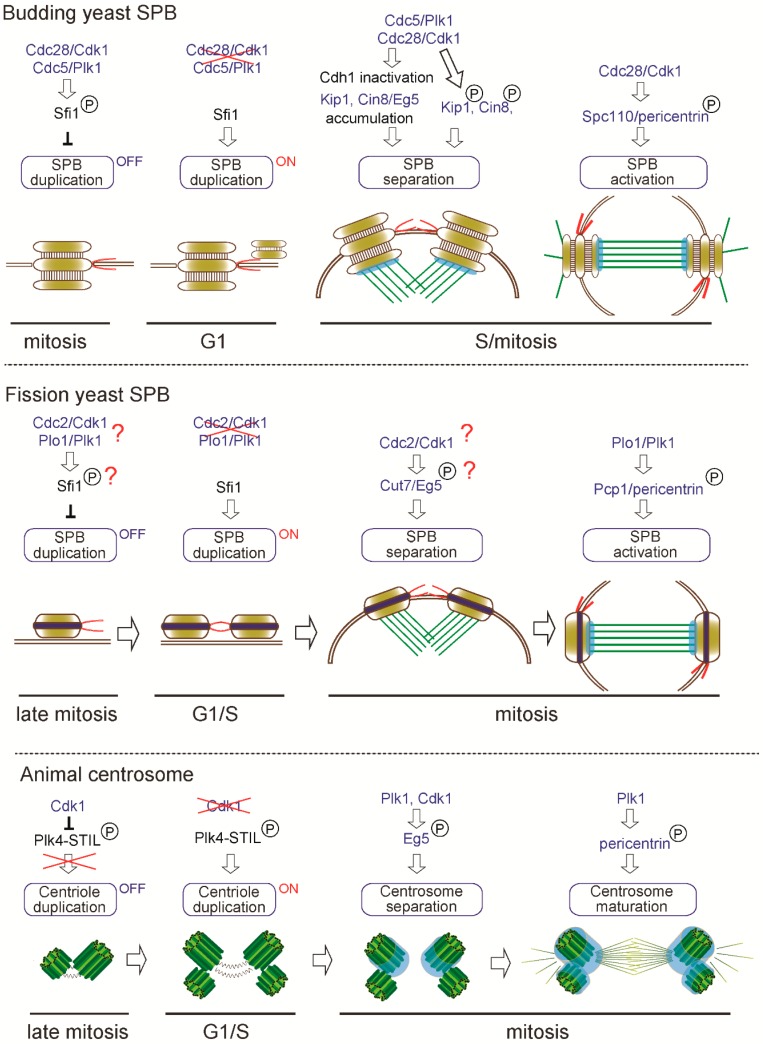
Common regulators of centrosome biogenesis and function. Similar regulators are involved in centrosome biogenesis: (1) duplication; (2) separation and (3) maturation/activation in yeasts and animals.

The centrosome duplicates only once in the cell cycle to form two centrosomes and its biogenesis is tightly regulated by multiple factors to ensure the correct number [25,90]. Once the centrosome is duplicated, the duplicated centrosomes are separated, matured and contribute to a mitotic spindle formation to achieve faithful cell division. Since the details of this process have been comprehensively reviewed elsewhere [25,91,92], we highlight the common regulators in this review. Interestingly, we find that common factors regulate the centrosome cycle in animal and yeast centrosome as illustrated in Figure 3. Here we focus on and overview the following critical steps: (i) centrosome duplication; (ii) separation; and (iii) maturation.

One century ago, the founder of centrosome biology, Boveri, proposed that increased number of centrosomes could lead to cancer [93] and it is known that indeed centrosome aberrations contribute to human diseases including cancer and microcephaly [94,95,96]. Both in animal and yeasts, cells have a mechanism to ensure the correct centrosome number. In the budding yeast *S. cerevisiae*, it is reported that the SPB duplication is limited to once in cell cycle by phospho-regulation of Sfi1, a half bridge component [97,98]. As illustrated in Figure 3, Cdc28/Cdk1 (cyclin-dependent kinase 1) and Cdc5/Plk1 (polo-like kinase) phosphorylate the serine/threonine residues in the C-terminus of Sfi1, thereby blocking the initiation of SPB duplication during S phase to early mitosis (“OFF” state). After late mitosis to G1, as Cdk1 and Plk1 are inactivated and the Cdc14 phosphatase dephosphorylates Sfi1, SPB duplication is licensed (“ON” state) and the new SPB is assembled on the half bridge.

Importantly, the fission yeast *S. pombe* Sfi1 contains only one Cdk1 consensus site and six putative Plk1 phosphorylation sites in the C-terminus although it is not known whether the phospho-regulation is critical for SPB duplication similarly as in *S. cerevisiae* [91]. 

While SPB duplication is dependent on the conserved Sfi1-centrin3 complex, the initiation of centriole duplication in animals is controlled by Plk4, a serine-threonine kinase acting as a master regulator of centriole biogenesis in animals [71,99,100]. Active PLK4 is known to recruit and phosphorylate its binding protein STIL, which then recruits SAS-6 and initiates centriole formation [101,102,103,104]. Importantly, Plk4 is lost from the genomes of yeasts but is present in the basal fungi Chytrid [7], suggesting that centriole biogenesis was regulated at the centrosome of the common ancestor of animal and fungi. Although animals and yeasts use distinct modules for centriole/SPB duplication, analogous phospho-regulation seems to play critical roles in limiting the timing of duplication. It was previously shown that inhibition of Cdk1 in multiple animal systems (CHO cells, chicken DT40 cells and *Drosophila* wing disc) leads to unscheduled centriole formation and amplification [105,106]. Recent studies revealed that Cdk1 inhibits premature centriole formation in mitosis by binding and phosphorylating STIL, thereby preventing STIL-PLK4 association, a critical initial step of centriole biogenesis [107,108] (Figure 3). Coinciding with inactivation of Cdk1 upon mitotic exit, it is thought that PLK4 binds and phosphorylates STIL in G1, allowing for the beginning of a centriole assembly in S phase [108] (“OFF” to “ON” state, Figure 3). Remarkably, the common kinase, Cdk1, regulates the timing of SPB/centriole duplication in both yeasts and animals. 

Once centrosome duplication is complete, separation of the two centrosomes is indispensable to assemble a bipolar spindle during mitosis. In *S. cerevisiae*, SPB separation is triggered by severing the bridge between two duplicated SPBs. This process is driven by the kinesin-5 motor proteins (Eg5 orthologue), Cin8 and Kip1, that create a pushing force to separate the two SPBs [109,110,111]. Adequate accumulation of the two kinesins requires inactivation of the anaphase-promoting complex (APC) activator Cdh1 through its phosphorylation by both Cdk1 and Plk1 [110,111]. In addition to the indirect effect, Cdk1 directly phosphorylates both Cin8 and Kip1 in vitro and in particular, phosphorylation on the conserved CDK consensus site (S/T-P-X-X) (S388) in the motor domain of Kip1 is required for SPB separation in vivo [112]. Cdk1 phosphorylation on the C-terminus of Sfi1 is also important for bridge severing and SPB separation [97].

In *S. pombe*, the sole kinesin-5 Cut7 (Eg5 orthologue) associates with the SPB and the mitotic spindle and is essential for SPB separation and bipolar spindle assembly similarly as *S. cerevisiae* Kip1 and Cin8 [113,114]. The above-mentioned CDK consensus site in the motor domain is conserved among kinesin-5 family proteins from yeast to human, however, it is not tested if this site affects the function of the protein in other species. Although it is not known if Cdc2/Cdk1 phosphorylates Sfi1 in *S. pombe*, the Sfi1-binding protein Cdc31/centrin3 is phosphorylated by Cdc2/Cdk1 and this phosphorylation is required for the partial dissociation of Sfi1 and timely SPB separation [66].

Similarly, as in yeasts, the kinesin-5 (Eg5) is required for centrosome separation and bipolar spindle formation in animals and Cdk1-phosphorylation on the residue in the C-terminal tail domain (T927) is shown to be required for association with the centrosome and mitotic spindle [115,116]. Another study revealed that centrosome separation occurs in Cdk1-inhibited DT40 cells and other Cdks and Plk1 also collaborate to trigger centrosome localization of Eg5 and centrosome separation [117]. Collectively, Eg5-dependent centrosome separation is phospho-regulated by Cdk1 and/or Plk1 activity and this force-driven mechanism is conserved despite the structural divergence of centrosomes in animal and yeasts (Figure 3).

In addition to the Eg5-dependent mechanism, centrosome separation is also achieved by severing the proteinaceous linker between the duplicated centrosomes in animal cells. Briefly, the linker containing the proteins such as C-Nap1 and rootletin is dissociated by the activity of kinases such as Nek2A, Plk1 and Aurora A at the G2/M boundary of the cell cycle (refer to the details of the molecular pathways in the reviews such as [92,118,119,120]). 

As the two centrosomes successfully separate to opposite sides, a bipolar spindle is assembled to achieve faithful chromosome segregation and centrosome inheritance. Since the mechanism for mitotic spindle assembly is comprehensively summarized in recent reviews such as [121,122], we will highlight the key phospho-regulation at the centrosome involved in mitotic spindle formation which is common in animal and yeasts in this review. 

As we described above, yeasts and animals share the PCM modules such as pericentrin and CDK5RAP2 and these proteins anchor γ-TuSC/γ-TuRC to the SPB/centriole. The microtubule-nucleating activity of the centrosome changes during the cell cycle, peaking at mitosis. This process is referred to as SPB activation in yeasts [123] and centrosome maturation in animals [124,125].

In *S. cerevisiae*, the γ-TuSC receptor Spc110/pericentrin interacts with γ-TuSC through its N-terminus and phosphorylation at this region by Cdk1 and Mps1 kinases promotes the affinity to γ-TuSC and oligomerization of γ-TuSC [47]. This phosphorylation is cell-cycle dependent, peaking in cells arrested in mitosis and allowing mitotic spindle assembly (Figure 3). In *S. pombe*, the corresponding Pcp1/pericentrin also recruits γ-TuRC presumably through the N-terminal conserved γ-TuSC/γ-TuRC binding motifs [47,62]. Pcp1 is also phosphorylated during mitosis partly in a Plk1-dependent manner and this phosphorylation is required for Pcp1 incorporation into the SPB [126]. Although phosphorylation sites have not been mapped yet, it is possible that Plk1-dependent phosphorylation might promote γ-TuRC targeting to drive mitotic spindle assembly from the SPBs during mitosis.

In animals, at the onset of mitosis, Plk1 plays a critical role in centrosome maturation as revealed by depletion or chemical inhibitor treatment [127,128,129]. Similarly, as in yeasts, it is shown that pericentrin is phosphorylated by Plk1 in mitosis and the phosphorylation on the residues conserved among vertebrates is required for accumulation of the PCM proteins such as CEP192 and GCP-WD, γ-tubulin and Plk1 [130]. Intriguingly, phospho-regulation of pericentrin to achieve higher microtubule-nucleating activity is also conserved in yeasts and animals despite the structural diversification in pericentrin protein sequences [46,47]. Taken together, we can find multiple conserved regulations in the analogous processes of centrosome biogenesis as illustrated in Figure 3, suggesting that regulators such as kinases and motors remained associated with the centrosome structure along evolution.

## 5. How Did the SPB Evolve?

As we compared above, the animal centrosome and the yeast SPBs show the great differences in morphology but some molecular components and regulation are conserved. Importantly, while the yeast acquired specific SPB components, the PCM is conserved. Furthermore, the timing and regulation of centrosome biogenesis amongst canonical centrosomes and SPBs are quite similar (Figure 3). It is likely that the ancestors of fungi are aquatic forms with flagellated spores and the loss of centrioles seems to have coincided with innovations in spore dispersal, that is, wind-dispersed non-flagellated spores found in extant yeasts [131]. It is thus possible that concomitant with loss of the centriole in yeasts, a new scaffold that harbours molecules that nucleate MTs and regulate signalling arose while maintaining similar functions. How did such structural transition occur in evolution? Intriguingly, the zygomycete SPB with a centriole-like structure provides hints about the evolutionary process [15]. This observation raises the possibility that the SPB arose “on” the pre-existing centriole at the centrosome. It still remains unknown how yeasts gained the SPB. Fission yeast SPB is composed of not only the conserved PCM components but also the proteins with no clear orthologues outside of fungi such as Ppc89 and Sid4 [63,64], suggesting acquisition of new proteins after branching to yeasts.

**Figure 4:**
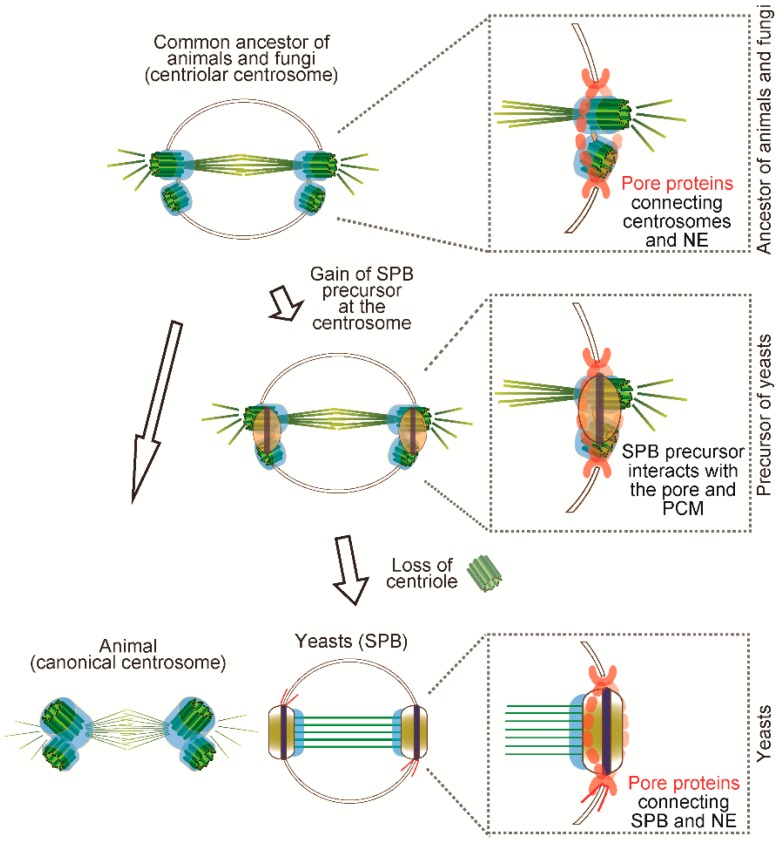
Speculated scenario of centrosome evolution. The common ancestor of fungi and animals is likely to have had a canonical centrosome. Presumably, it might be like the extant chytrid centrosome in which the centriole pair is anchored in the NE to undergo closed mitosis. Eventually, the newly invented SPB precursor was formed closed to the centriolar centrosome and started to interact with the pore and PCM. When the centriole was lost, the SPB structure replaced the role of centrioles and maintained the same function as MTOC.

By inferring from the extant chytrid centrosome, it is likely that the animal-fungi ancestor might have had a centrosome with a pair of centrioles anchored in the nucleus which underwent closed mitosis (Figure 4). We propose that the yeast SPB has been formed possibly by step-wise processes from the common ancestor. As the first step, the SPB precursor appeared on the ancestral centriolar centrosome and then it interacted with the existing PCM proteins and connected to the NE. Finally, when centriole and cilia were lost from the genome, the SPB precursor replaced the roles of centrioles as a scaffold for PCM modules (Figure 4). Even after yeasts lost the centriole and the function for motility, the necessity to maintain the other function, that is, microtubule nucleation, might have constrained the evolution of PCM structure and function.

## 6. Conclusions and Perspective

In the field of centrosome biology, the yeast SPB has been described as “equivalent to the centrosome” but its evolution and origin have been less understood and discussed. In this review, we speculate that the animal centrosome and the yeast SPBs are indeed homologous since they share the similar structure and molecules originated from the common ancestor. In particular, the PCM is remarkably conserved in terms of molecular composition and regulation due to its indispensable function to nucleate microtubules at the centrosome. As we discussed above, analyses of the centrosome structure, components and functions from a greater variety of species give us more hints in order to reconstitute the evolutionary history of the centrosome and understand how the ancestral centrosome was remodelled and diverse structures arose. Better understanding the centrosome evolution will lead to discovering the essential and fundamental mechanism to assemble the functional organelle. The diverged centrosomes should continue to be a great model to understand how the centrosomes evolved and biogenesis is regulated.
